# Aerosol Inhalation of a Recombinant H7N9 Hemagglutinin Antigen Elicits Systemic and Mucosal Immune Responses in Mice

**DOI:** 10.3390/v18050579

**Published:** 2026-05-21

**Authors:** Zhuoran Hou, Han Wang, Bin Zhang, Ruixi Liu, Yuli Zhang, Ye Yang, Jianxin Wu, Xuchen Hou, Xiuguo Ge, Jun Wu, Bo Liu

**Affiliations:** 1 Tianjin Key Laboratory of Agricultural Animal Breeding and Healthy Husbandry, College of Animal Science and Veterinary Medicine, Tianjin Agricultural University, Tianjin 300392, China; houzhuoranzzz@126.com; 2 National Key Laboratory of Advanced Biotechnology, Academy of Military Medical Sciences, Beijing 100071, China; wanghan010811@163.com (H.W.); zhangbin@bmi.ac.cn (B.Z.); liuruixi0521@outlook.com (R.L.); zyl1024370@163.com (Y.Z.); yangye012023@163.com (Y.Y.); w13261283975@163.com (J.W.); hoxuch@163.com (X.H.); 3 College of Animal Science and Technology, Northwest A&F University, Yangling 712100, China; 4 School of Life Sciences and Medical Engineering, Anhui University, Hefei 230601, China

**Keywords:** A(H7N9) influenza, hemagglutinin, yeast expression, mucosal vaccination, immunogenicity

## Abstract

Highly pathogenic avian influenza A (H7N9) remains a threat to poultry health and poses a zoonotic risk, highlighting the need for vaccine antigens capable of inducing both systemic and mucosal immunity. In this study, we evaluated X33CLS-H7, a clarified cell-lysate supernatant derived from glycoengineered *Pichia pastoris* expressing H7 hemagglutinin, in BALB/c mice following intramuscular(i.m.) injection, nebulized inhalation, or intranasal instillation. H7 expression and hemagglutination activity were confirmed by Western blotting and hemagglutination assay, respectively. Serum HA7-specific IgG and IgA responses, hemagglutination inhibition(HI) activity, H7N9 pseudovirus neutralization, bronchoalveolar lavage fluid (BALF) antibodies, and safety readouts were assessed. After two i.m. immunizations, X33CLS-H7 induced the strongest systemic antibody responses, with an HI geometric mean titer of 1:1622 95% CI, 1:1108–1:2348 and a mean log10 NT_50_ of 4.62. Respiratory immunization also elicited antibody responses. After four doses, high-dose nebulized delivery produced the strongest responses among the respiratory delivery regimens, with serum IgG and IgA titers of 1.02 × 10^5^ and 2.24 × 10^3^, respectively, an endpoint HI GMT r of 1:457 95% CI, 1:211–1:971, and a mean log10 NT_50_ of 3.77 compared with 2.02 in saline controls. High-dose nebulized delivery also generated detectable HA7-specific IgG and IgA responses in bronchoalveolar lavage fluid. No overt local or systemic toxicity signals were observed under the tested conditions. These findings indicate that X33CLS-H7 retains HA7-associated antigenicity and can induce systemic and respiratory mucosal antibody responses, supporting its further evaluation as a simplified and scalable H7N9 vaccine antigen candidate.

## 1. Introduction

Highly pathogenic avian influenza A viruses, particularly the H5 and H7 subtypes, continue to circulate and evolve rapidly in poultry, enabling efficient transmission through respiratory secretions and aerosolized particles and posing persistent threats to animal health, poultry production, and public health [1,2]. The emergence of human H7N9 infections since 2013 has further highlighted that avian epidemic strains not only cause substantial losses in the poultry industry but can also cross the species barrier and result in severe human disease [3,4,5]. Accordingly, reducing infection establishment and viral shedding at the poultry level represents an important upstream intervention that may benefit both animal and public health.

Current influenza vaccines, particularly conventional intramuscular inactivated or subunit vaccines, are generally effective at inducing systemic IgG and hemagglutination inhibition (HI)-associated antibody responses, and HI has long been used as an established correlate of protection [6]. However, such responses do not necessarily indicate the establishment of an adequate secretory IgA barrier or local mucosal immune memory at the respiratory mucosa, which is the primary portal of infection. In addition, mucosal and peripheral immune responses show a certain degree of compartmentalization, meaning that serum readouts cannot simply substitute for local mucosal immunity [7]. By contrast, mucosal vaccination has the potential to induce secretory antibodies and tissue-resident T-cell responses at the site of pathogen entry, thereby blocking infection at an early stage and more closely aligning with the goal of reducing viral shedding and transmission [8].

In practice, however, the development of mucosal subunit vaccines remains challenging because protein antigens are often weakly immunogenic and must overcome multiple barriers, including limited mucosal uptake and an immunoregulatory local microenvironment [9]. Therefore, optimization of delivery strategies and establishment of more informative immune evaluation systems are particularly important for mucosal influenza vaccine development. Previous studies have shown that intranasal boosting with unadjuvanted HA protein can significantly enhance local respiratory immunity in mice and that nasal IgA levels are negatively associated with upper respiratory viral burden [10]. These findings support a close relationship between mucosal IgA, viral shedding duration, and infection risk. Accordingly, for next-generation influenza vaccines intended to address both protection and transmission interruption, respiratory mucosal delivery strategies, including nasal administration and aerosol inhalation, together with combined evaluation of systemic and local immune readouts, are becoming increasingly important [11,12].

In the present study, full-length H7 hemagglutinin was expressed in the glycoengineered *Pichia pastoris* X33-7 strain, and an antigen candidate, designated X33CLS-H7, was prepared in the form of clarified cell-lysate supernatant (CLS). This platform offers practical advantages for industrial production, including high-density fermentation, a relatively simple process, and lower manufacturing cost, while glycoengineering may help mitigate the potential impact of yeast-type high-mannose glycans on antigen conformation and consistency [13]. Previous studies have shown that full-length HA7 expressed in glycoengineered *Pichia* can undergo complex glycosylation, self-assemble into nanoparticle structures, induce HI activity, and confer protection in mice, thus providing a rationale for both the expression platform and antigen design used here [14]. On this basis, we systematically compared intramuscular immunization with respiratory mucosal delivery, including nebulized inhalation and intranasal instillation, in mice and evaluated their effects on serum IgG, serum IgA, HI activity, and bronchoalveolar lavage fluid (BALF) IgG and IgA responses. Overall tolerability was also assessed to provide experimental evidence for further optimization of H7 mucosal subunit vaccines aimed at reducing viral shedding and transmission.

## 2. Materials and Methods

### 2.1. Glycoengineered Yeast Strain and Reagents

The *HA* gene from A/Anhui/1/2013 (H7N9) was codon-optimized for yeast expression, cloned into the pPICZαA vector, and transformed into the glycoengineered *Pichia pastoris* X33-7 strain [15]. The HA construct retained its native transmembrane domain to facilitate membrane anchoring and help preserve relevant conformational and antigenic features.

The main materials and reagents used in this study were as follows. For yeast culture, yeast extract, agar, and peptone were purchased from OXOID (Basingstoke, UK), and NaCl was obtained from Sinopharm (Shanghai, China). Zeocin was purchased from Thermo Fisher Scientific (Waltham, MA, USA). Complete Freund’s adjuvant (CFA) and incomplete Freund’s adjuvant (IFA) were obtained from MedChemExpress LLC (Shanghai, China). AS03, an oil-in-water emulsion containing squalene and α-tocopherol, was used as an adjuvant. HRP-conjugated goat anti-mouse IgG and IgA were purchased from Abcam (Cambridge, UK). The anti-H7N9 hemagglutinin (HA) antibody and HRP-conjugated goat anti-rabbit IgG were obtained from Beijing Yiqiao Shenzhou Tech (Beijing, China).

### 2.2. Shake-Flask Induction and Preparation of X33CLS-H7 Antigen

Positive clones were first cultured in shake flasks containing YPD broth supplemented with 1 μg/mL Zeocin to expand biomass. The cultures were then diluted 1:20 (*v*/*v*) into buffered glycerol-complex medium (BMGY) for further cell growth. When the cultures reached the desired cell density, the cells were transferred to a methanol induction medium derived from BMGY and incubated at 25 °C with shaking for 48 h. To maintain induction, methanol was added every 24 h to a final concentration of 1% (*v*/*v*), for a total of two feeds.

After induction, the cells were harvested by centrifugation (8500× *g*, 20 min, 4 °C) and resuspended in 5 mM EDTA at a volume equivalent to 20% of the original culture volume. The cells were lysed by high-pressure homogenization at >750 bar for three passes at 4 °C. The lysate was clarified by centrifugation (8500× *g*, 20 min, 4 °C), and the resulting supernatant was collected as the antigen stock and designated X33CLS-H7.

X33CLS-H7 was intentionally prepared as a clarified cell-lysate supernatant rather than as a purified recombinant HA preparation. Accordingly, the preparation may contain residual yeast-derived components in addition to the target HA7 antigen. All procedures were performed on ice to minimize proteolytic degradation. The antigen preparation was aliquoted and stored at −80 °C until use.

### 2.3. X33CLS-H7 Antigen Characterization and HA Activity

The hemagglutination activity of X33CLS-H7 was determined using a red blood cell (RBC) agglutination assay. Briefly, samples were subjected to two-fold serial dilutions starting at 1:10 in V-bottom 96-well plates. An equal volume of 1% chicken RBC suspension was added to each well, gently mixed, and incubated at room temperature for 30 min. The highest dilution that showed complete hemagglutination was recorded as the hemagglutination titer, expressed as hemagglutination units (HAU).

In the present study, HAU was used primarily as a functional normalization metric for batch comparison and dose matching rather than as a direct biochemical quantification of absolute HA protein concentration. Therefore, the reported X33CLS-H7 doses should be interpreted as estimated HA-equivalent inputs rather than as absolute purified HA mass.

Western blotting was performed to qualitatively verify antigen expression using anti-H7N9 HA antibodies. A specific band at the expected molecular weight of approximately 75 kDa was detected, confirming the presence of the target HA7 in the X33CLS-H7 preparation.

### 2.4. Experimental Design and Immunization Regimens

Female BALB/c mice (6–8 weeks old, specific pathogen-free [SPF]) were purchased from Beijing Vital River Laboratory Animal Technology Co., Ltd. (Beijing, China) and housed under SPF barrier conditions. All animal procedures were approved by the Institutional Animal Care and Use Committee (IACUC; approval no. IACUC-2024-042).

To evaluate the effects of different delivery routes on immune responses, mice were randomly assigned to the following groups (*n* = 6 per group): X33CLS-H7_i.m._ (intramuscular administration, two doses on days 0 and 14); X33CLS-H7_NE-high_ (high-dose nebulized inhalation, four doses on days 0, 14, 28, and 42); X33CLS-H7_NE-low_ (low-dose nebulized inhalation, four doses); rH7_NE-low_ (low-dose nebulized inhalation of purified rH7, four doses, with purified rH7 administered at an estimated HA-equivalent input matched to that of the X33CLS-H7_NE-low_ group); X33CLS-H7_i.n.-high_ (high-dose intranasal instillation, four doses); X33CLS-H7_i.n.-low_ (low-dose intranasal instillation, four doses); an intramuscular host-strain lysate control group (Control); and a nebulized saline control group (Saline). The vaccine dose and administration volume for each group are summarized in Table 1.

For nebulized immunization, the stated dose refers to the amount loaded into the nebulizer system rather than a directly measured deposited respiratory dose. A higher nominal loading was used for nebulization because only a fraction of the aerosolized formulation is expected to be effectively inhaled and deposited in the respiratory tract owing to device dead volume, chamber retention, aerosol dispersion, and exhalation. For intranasal instillation, the high- and low-dose regimens were generated by administering different total volumes from the same antigen stock preparation. Thus, in the present design, dose and volume effects were not fully separable for the intranasal groups.

To assess the effects of different adjuvants under a single-dose intramuscular regimen, X33CLS-H7 was mixed 1:1 (*v*/*v*) with CFA, IFA, or AS03 and administered intramuscularly once. Because 1:1 mixing reduced the antigen dose by half, a 9 μg intramuscular no-adjuvant group was included as a dose-matched antigen control, while the 18 μg intramuscular group was included as a reference. In addition, a CFA-only control group without X33CLS-H7 was included to monitor adjuvant-associated background effects, because CFA was considered the most reactogenic adjuvant among those tested. Adjuvant-only controls were not established for every adjuvant in the present design. A concise summary of the experimental groups, immunization regimens, and principal evaluation readouts is provided in Table 2.

Before immunization, the mice were acclimated and monitored to ensure good health status. For intramuscular injection, formulations were administered into the thigh muscle of the hind limb using a 0.5 mL syringe, and each injection was completed within approximately 10 s to minimize stress. Nebulized immunization was performed using a small-animal nebulizer (YLS-8B08; Beijing Yingze Tonghui Biotechnology, Beijing, China) set to level 2. Each mouse was placed individually in the chamber, and aerosolization was maintained for approximately 10 min per dose. For intranasal immunization, the indicated volume of antigen preparation was administered dropwise into the nostrils to allow spontaneous inhalation.

### 2.5. Hemagglutination Inhibition (HI) Assay

HI assays were performed according to the World Health Organization (WHO)-recommended microtiter protocol. Serum samples were treated with receptor-destroying enzyme (RDE) by mixing the serum with RDE at a 1:3 (*v*/*v*) ratio, followed by incubation at 37 °C overnight (16–18 h). The samples were then heat-inactivated at 56 °C for 30 min to inactivate complement.

Treated sera were subjected to two-fold serial dilutions starting at 1:10 in V-bottom 96-well plates. The diluted sera were then mixed with an equal volume of standardized antigen containing 4 HAU of HA7, incubated, and subsequently combined with 1% chicken erythrocytes. HA7 was a purified glycoengineered yeast-derived rH7 antigen derived from the same HA7 sequence as X33CLS-H7, namely A/Anhui/1/2013 (H7N9), and was used here as the standardized HI antigen.

The HI endpoint titer for each sample was defined as the highest serum dilution that completely inhibited hemagglutination. Individual HI endpoint titers were determined by conventional two-fold serial dilution, whereas group-level data are presented as geometric mean reciprocal titers (GMTs) calculated from the individual endpoint values. An HI titer of ≥1:40 was used as a reference threshold indicative of substantial hemagglutination-inhibiting antibody activity, consistent with previous reports [16]. HI GMTs and corresponding 95% confidence intervals were calculated from log10-transformed individual HI endpoint titers and then back-transformed to the reciprocal titer scale. Samples with HI titers below the lower limit of detection were assigned a reciprocal titer of 10 for calculation.

### 2.6. ELISA for HA7-Specific IgG and IgA in Serum and BALF

High-binding ELISA plates (3590, Corning, NY, USA) were coated overnight at 4 °C with purified rH7 (2 μg/mL) in carbonate coating buffer (50 mmol/L, pH 9.6). Purified rH7 was prepared in our laboratory based on the same HA7 sequence as X33CLS-H7 (A/Anhui/1/2013 [H7N9]). This coating strategy was intended to facilitate detection of HA7-specific antibody binding while minimizing direct reactivity to residual components in the clarified lysate preparation. Purified rH7 therefore served as a standardized HA7-specific coating antigen in the ELISA. The plates were washed three times with PBST (PBS containing 0.05% Tween-20) and then blocked with 5% (*w*/*v*) non-fat milk in PBST for 1 h at 37 °C. To further reduce potential background reactivity to yeast-derived components, mouse serum samples were preincubated with X33 yeast lysate at 4 °C for 30 min before dilution. The samples were then serially diluted starting at 1:50, added to the plates, and incubated for 1 h at 37 °C. After three washes, HRP-conjugated goat anti-mouse IgG (1:5000) was added and incubated for 1 h at 37 °C. The plates were washed, developed with a one-component TMB substrate (PR1200, Solarbio, Beijing, China) for 4 min, stopped with 2 M H_2_SO_4_, and the absorbance at 450 nm was measured using a SpectraMax iD3 multimode microplate reader (Molecular Devices, Shanghai, China). The ELISA endpoint titer was defined as the highest reciprocal dilution yielding an OD450 value exceeding 2.1 × blank. Samples with titers below the starting dilution were assigned the reciprocal value of the starting dilution (1:50) for plotting and statistical analysis; accordingly, these samples appear as log10(50) = 1.70 in the graphs.

For IgA detection, the same procedure was followed except that HRP-conjugated goat anti-mouse IgA was used. In addition to serum antibody detection, bronchoalveolar lavage fluid (BALF) collected from the X33CLS-H7_NE-high_ group and the saline control group two weeks after the final immunization was also analyzed for HA7-specific IgG and IgA. BALF analysis was restricted to the high-dose nebulized group because evaluation of lower-airway antibody induction under aerosol delivery was prioritized in the present study.

### 2.7. Pseudovirus Neutralization Assay

Neutralizing antibody activity against H7N9 was evaluated using an HIV-1-based H7N9 pseudovirus neutralization assay. Briefly, Influenza A H7N9 (A/Anhui/1/2013) pseudovirus (PSVD33; Sino Biological, Beijing, China), carrying a luciferase reporter gene, was activated with TPCK-treated trypsin at a concentration of 100 μg/mL and incubated at 37 °C for 1 h before use. Serum samples were heat-inactivated and serially diluted. Equal volumes of diluted sera and pseudovirus were mixed and incubated at 37 °C for 1 h to allow antibody–pseudovirus interaction.

The serum–pseudovirus mixtures were then added to 293T cells seeded in 96-well plates, with 50 μL pseudovirus used per well. After incubation at 37 °C for 48 h, luciferase activity was measured using a SpectraMax iD3 multimode microplate reader (Molecular Devices, Shanghai, China) to determine pseudovirus infection. Neutralization titers were expressed as the 50% neutralization titer (NT_50_), defined as the reciprocal serum dilution that reduced the luciferase signal by 50% relative to the virus-only control. NT_50_ values were log10-transformed for statistical analysis and graphical presentation.

### 2.8. Body Weight Monitoring and Post-Immunization Observations

All mice were monitored daily for general health status and adverse events. Body weight was recorded longitudinally from baseline, and the percentage change from baseline was calculated to identify potential toxicity signals, such as weight loss or growth retardation, associated with the different formulations or delivery routes. Post-immunization observations included coat condition, activity level (e.g., lethargy or reduced activity), respiratory signs (e.g., nasal discharge, with particular attention to the NE and i.n. groups), and injection-site reactions in the i.m. groups (e.g., erythema, swelling, and induration). All abnormal findings were recorded with their corresponding time points.

### 2.9. Histopathology

To evaluate the potential local tissue effects of mucosal immunization, such as airway inflammation or injury, as well as the systemic effects of intramuscular immunization, mice in the X33CLS-H7_i.m._, X33CLS-H7_NE-high_, and saline control groups were euthanized two weeks after the final immunization for histopathological examination. The heart, liver, spleen, lung, and kidney were collected, fixed in 4% paraformaldehyde, and processed for hematoxylin and eosin (H&E) staining by a commercial service provider (Servicebio, Wuhan, China).

### 2.10. Serum Biochemistry

Two weeks after the final immunization, blood samples were collected from mice in the X33CLS-H7_i.m._, X33CLS-H7_NE-high_, and saline control groups for serum biochemistry analysis. Liver-associated markers, including AST, ALT, and ALP, as well as the renal marker BUN, were quantified using an automated biochemical analyzer to assess potential systemic toxicity and organ stress. The measured values were compared with those of the control mice.

### 2.11. Statistical Analysis

GraphPad Prism 10.1.2 (GraphPad Software, Boston, MA, USA) was used for data visualization and statistical analysis. Paired *t*-tests were used for within-group comparisons across different time points, and unpaired *t*-tests were used for between-group comparisons, as appropriate. Statistical significance was defined as follows: ns, *p* ≥ 0.05; * *p* < 0.05; ** *p* < 0.01; *** *p* < 0.001; and **** *p* < 0.0001. Data are presented as the mean ± standard deviation (SD).

## 3. Results

### 3.1. Expression of X33CLS-H7 and Associated Hemagglutination Activity

X33CLS-H7 was prepared and evaluated as a clarified cell-lysate supernatant rather than as a purified recombinant HA product. Because this antigen format contains abundant host-derived background proteins, conventional SDS-PAGE analysis was not used for purity assessment. Instead, Western blotting was performed to qualitatively verify the presence of the target HA7 in the X33CLS-H7 preparation before subsequent immunogenicity evaluation.

**Figure 1:**
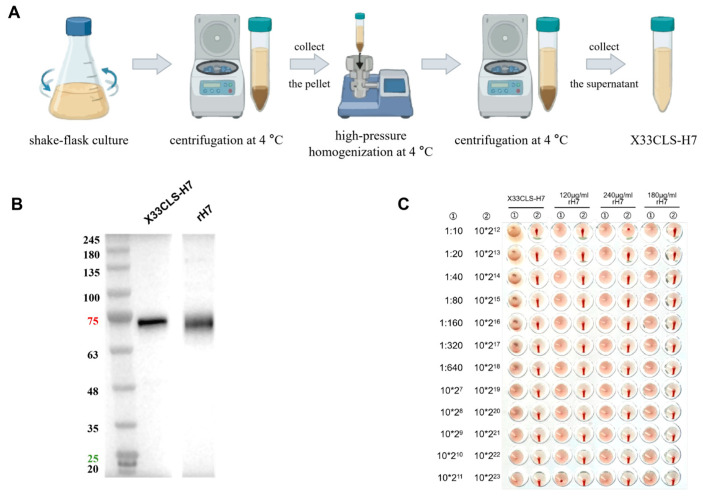
Preparation and characterization of X33CLS-H7. (**A**) Schematic illustration of the preparation workflow for X33CLS-H7. (**B**) Western blot analysis of X33CLS-H7 using an HA7-specific antibody. Purified rH7 was included as a reference control. (**C**) Hemagglutination assay of X33CLS-H7 and reference rH7 preparation.

Western blot analysis using an HA7-specific antibody revealed a distinct band at the expected molecular weight of approximately 75 kDa, confirming the presence of target HA7 in the X33CLS-H7 preparation (Figure 1B). To support batch-to-batch comparison and dose normalization, hemagglutination activity was further assessed using a chicken erythrocyte hemagglutination assay. The results showed detectable hemagglutination activity in the X33CLS-H7 preparation, and HAU was therefore used as a functional readout of relative HA-associated activity for subsequent dose estimation (Figure 1C).

### 3.2. Intramuscular Immunization with X33CLS-H7 Induced Robust Antibody Responses

**Figure 2:**
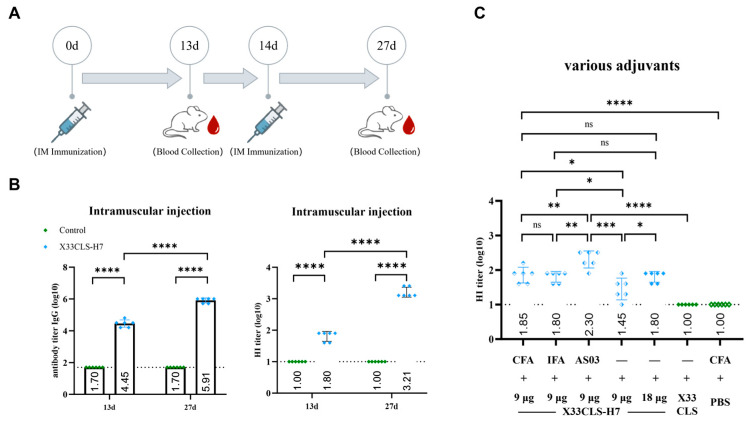
Intramuscular immunization with X33CLS-H7 induced strong systemic antibody responses. (**A**) Schematic diagram of the intramuscular immunization schedule. Mice were immunized on days 0 and 14, and blood samples were collected on days 13 and 27. (**B**) Serum HA7-specific IgG titers and hemagglutination inhibition (HI) titers in mice after intramuscular immunization with X33CLS-H7, compared with the control group, at the indicated time points. (**C**) Comparison of HI titers induced by X33CLS-H7 formulated with different adjuvants after a single intramuscular immunization. CFA, IFA, and AS03 were evaluated, together with no-adjuvant antigen controls, and a CFA-only control condition, as indicated. In ELISA panels, titers are shown as log10 reciprocal endpoint titers. Values below the starting dilution were assigned 1:50 (log10 = 1.70) for plotting. In the HI panels, the dotted line indicates the lower limit of detection (1:10). Data are presented as mean ± SD. Statistical analysis was performed using paired *t*-tests for within-group comparisons across time points and unpaired *t*-tests for between-group comparisons, as appropriate. Statistical significance is shown in the figure (* *p* < 0.05, ** *p* < 0.01, *** *p* < 0.001, **** *p* < 0.0001; ns, not significant).

Two intramuscular (i.m.) immunizations with X33CLS-H7 induced strong systemic antibody responses. Serum HA7-specific IgG endpoint titers increased markedly after immunization, reaching a group mean titer of 8.13 × 10^5^ after the second dose. Consistently, hemagglutination inhibition (HI) titers also increased after the second i.m. immunization, with the endpoint HI GMT reaching 1:1622 with a 95% CI of 1:1108–1:2348 at day 27, indicating efficient induction of hemagglutination-inhibiting antibodies (Figure 2B).

To assess whether adjuvant co-formulation could further enhance HI responses after a single intramuscular immunization, X33CLS-H7 was mixed with CFA, IFA, or AS03 and administered as a single i.m. dose. HI responses differed among the adjuvant groups, with AS03 eliciting higher titers than CFA or IFA (*p* < 0.01). CFA and IFA also increased HI responses compared with the no-adjuvant controls (*p* < 0.05), whereas no significant difference was observed between the CFA and IFA groups (Figure 2C).

The endpoint HI GMTs and corresponding 95% confidence intervals for all intramuscular immunization groups are summarized in Table 3.

### 3.3. Respiratory Mucosal Delivery Induced Systemic Antibody Responses and Dose-Dependent HI Activity

**Figure 3:**
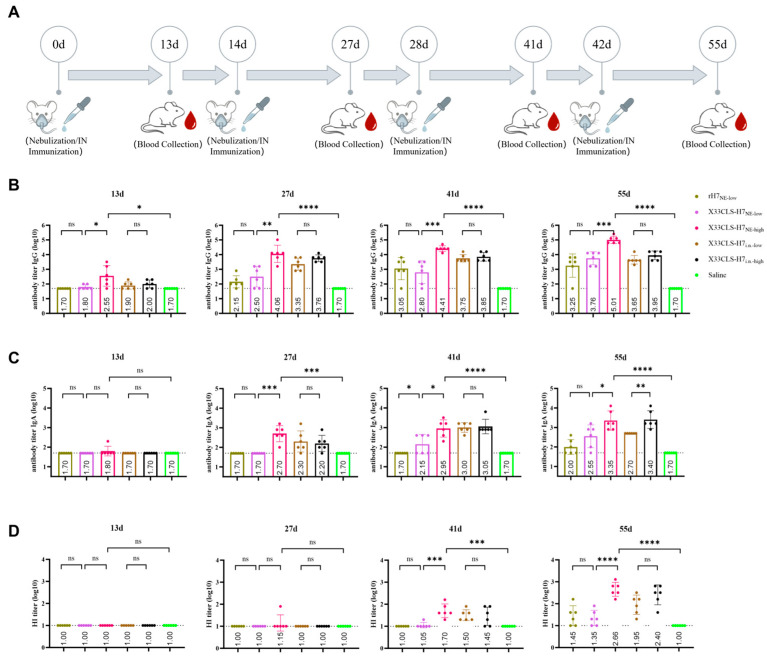
Respiratory mucosal immunization with X33CLS-H7 induced systemic antibody responses in mice. (**A**) Schematic diagram of the respiratory mucosal immunization schedule. Mice received nebulized inhalation or intranasal instillation on days 0, 14, 28, and 42, and blood samples were collected on days 13, 27, 41, and 55. (**B**) Serum HA7-specific IgG titers at the indicated time points in mice immunized with different respiratory mucosal regimens. (**C**) Serum HA7-specific IgA titers at the indicated time points. (**D**) Serum hemagglutination inhibition (HI) titers at the indicated time points. In ELISA panels, titers are shown as log10 reciprocal endpoint titers. Values below the starting dilution were assigned 1:50, corresponding to log10 = 1.70, for plotting. In HI panels, the dotted line indicates the lower limit of detection, 1:10. Data are presented as mean ± SD. Statistical analysis was performed using paired *t*-tests for within-group comparisons across time points and unpaired *t*-tests for between-group comparisons, as appropriate. Statistical significance is shown in the figure (* *p* < 0.05, ** *p* < 0.01, *** *p* < 0.001, **** *p* < 0.0001; ns, not significant).

Respiratory mucosal immunization with X33CLS-H7 elicited measurable systemic antibody responses in mice. In the high-dose nebulized group (X33CLS-H7_NE-high_), serum HA7-specific IgG titers were significantly higher than those in the saline control group after the first immunization (*p* < 0.05), and were also significantly higher than those in the low-dose nebulized group (X33CLS-H7_NE-low_; *p* < 0.05). After the second immunization, IgG titers in the X33CLS-H7_NE-high_ group increased further, and the difference from the X33CLS-H7_NE-low_ group became progressively more pronounced (Figure 3B). This advantage was maintained after subsequent immunizations, indicating a clear dose-associated enhancement of systemic IgG induction under nebulized delivery.

Serum HA7-specific IgA responses showed a broadly similar pattern. After the first immunization, no obvious differences were observed among the groups. However, after the second immunization, IgA titers in the X33CLS-H7_NE-high_ group became significantly higher than those in both the X33CLS-H7_NE-low_ group and the saline control group (*p* < 0.01) (Figure 3C). After the third and fourth immunizations, the X33CLS-H7_NE-high_ group continued to maintain higher IgA responses, suggesting that increasing the nebulized dose enhanced systemic IgA induction.

HI activity remained generally low after the first two immunizations but increased clearly after the third immunization. At day 41, the X33CLS-H7_NE-high_ group showed significantly higher HI titers than both the X33CLS-H7_NE-low_ group and the saline control group (*p* < 0.001). By day 55, this difference became more pronounced (*p* < 0.0001) (Figure 3D).

After four immunizations, the X33CLS-H7_NE-high_ group showed the strongest systemic antibody responses among the nebulized regimens, with serum HA7-specific IgG and IgA titers reaching approximately 1.02 × 10^5^ and 2.24 × 10^3^, respectively, and an endpoint HI GMT of 1:457 with a 95% CI of 1:211–1:971. In contrast, the low-dose nebulized group (X33CLS-H7_NE-low_) showed weaker responses, with serum IgG and IgA titers of approximately 5.75 × 10^3^ and 3.55 × 10^2^, respectively, and an endpoint HI GMT of 1:22 with a 95% CI of 1:10–1:53. These results further support a dose-associated increase in systemic antibody and hemagglutination-inhibiting responses under nebulized delivery.

Under matched low-dose nebulization, some differences were observed between the purified rH7_NE-low_ and X33CLS-H7_NE-low_ groups at intermediate time points (day 41). However, at the endpoint after four immunizations (day 55), no statistically significant differences were detected in serum HA7-specific IgG, IgA, or HI titers between the two groups (Figure 3B–D), suggesting comparable endpoint systemic immunogenicity between purified rH7 and X33CLS-H7 under these matched nebulized conditions.

The endpoint HI GMTs and corresponding 95% confidence intervals for all respiratory mucosal immunization groups at day 55 are summarized in Table 4.

**Figure 4:**
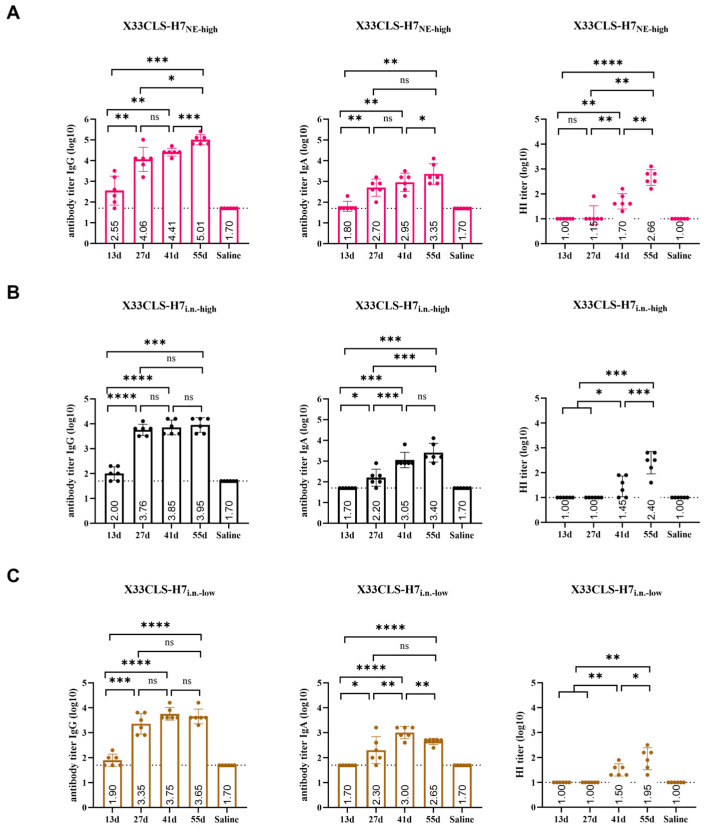
Kinetics of systemic antibody responses induced by representative high- and low-dose respiratory immunization regimens. (**A**) Time-course analysis of serum HA7-specific IgG titers, serum HA7-specific IgA titers, and HI titers in the X33CLS-H7_NE-high_ group on days 13, 27, 41, and 55, with the saline group shown as the negative control. (**B**) Time-course analysis of serum HA7-specific IgG titers, serum HA7-specific IgA titers, and HI titers in the X33CLS-H7_i.n.-high_ group. (**C**) Time-course analysis of serum HA7-specific IgG titers, serum HA7-specific IgA titers, and HI titers in the X33CLS-H7_i.n.-low_ group. In ELISA panels, titers are shown as log10 reciprocal endpoint titers. Values below the starting dilution were assigned a value of 1:50, corresponding to log10 = 1.70, for plotting. In the HI panels, the dotted line indicates the lower limit of detection, 1:10. Data are presented as mean ± SD. Statistical analysis was performed using paired *t*-tests for within-group comparisons across time points and unpaired *t*-tests for between-group comparisons, as appropriate. Statistical significance is shown in the figure (* *p* < 0.05, ** *p* < 0.01, *** *p* < 0.001, **** *p* < 0.0001; ns, not significant).

Intranasal instillation also induced systemic antibody responses, with broadly similar temporal trends to those observed under high-dose nebulized delivery (Figure 4A–C). After four immunizations, the X33CLS-H7_i.n.-high_ group achieved serum HA7-specific IgG and IgA titers of approximately 8.91 × 10^3^ and 2.51 × 10^3^, respectively, and an endpoint HI GMT of 1:254 with a 95% CI of 1:85–1:759. At the endpoint, no statistically significant differences were observed between the X33CLS-H7_i.n.-low_ and X33CLS-H7_i.n.-high_ groups in serum IgG titers or HI activity, whereas the high-dose intranasal group showed a higher IgA response than the low-dose intranasal group.

### 3.4. X33CLS-H7 Induced Neutralizing Antibodies Against H7N9 Pseudovirus

**Figure 5:**
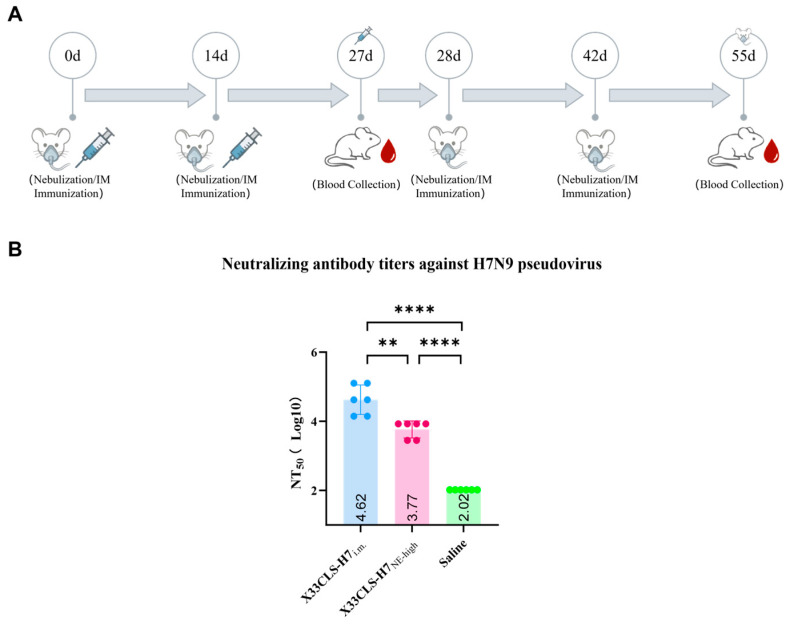
X33CLS-H7 induced serum neutralizing antibodies against H7N9 pseudovirus. (**A**) Schematic diagram of the immunization and serum collection schedule. Mice were immunized on days 0, 14, 28, and 42, and serum samples were collected on days 27 and 55 for the high-dose nebulized and saline control groups. (**B**) Serum neutralizing antibody titers against H7N9 pseudovirus were measured using a pseudovirus neutralization assay. Neutralizing titers are presented as log10 NT_50_ values. Data are shown as individual values with mean ± SD. Statistical analysis was performed using unpaired *t*-tests for between-group comparisons, as appropriate. Statistical significance is shown in the figure (** *p* < 0.01, **** *p* < 0.0001).

To further determine whether the antibody responses induced by X33CLS-H7 were capable of functionally blocking viral entry, serum samples were evaluated using an H7N9 pseudovirus neutralization assay. The influenza A H7N9 (A/Anhui/1/2013) pseudovirus carried a luciferase reporter gene, and NT_50_ was used as a functional readout of serum neutralizing activity. As shown in Figure 5, X33CLS-H7 immunization induced detectable neutralizing antibody responses against H7N9 pseudovirus.

Among the tested serum samples, the X33CLS-H7_i.m._ group showed a high neutralizing response, with a mean log10 NT_50_ value of 4.62. The X33CLS-H7_NE-high_ group also showed substantial neutralizing activity, with a mean log10 NT_50_ value of 3.77, which was significantly higher than that of the saline control group. In contrast, the saline group displayed only low background neutralizing activity, with a mean log10 NT_50_ value of 2.02. Statistical analysis showed that the NT_50_ titer in the X33CLS-H7_i.m._ group was significantly higher than that in the X33CLS-H7_NE-high_ group (*p* < 0.01), and both X33CLS-H7-immunized groups showed significantly higher neutralizing titers than the saline control group (*p* < 0.0001).

### 3.5. High-Dose Nebulization Induced Bronchoalveolar IgG and IgA Responses

**Figure 6:**
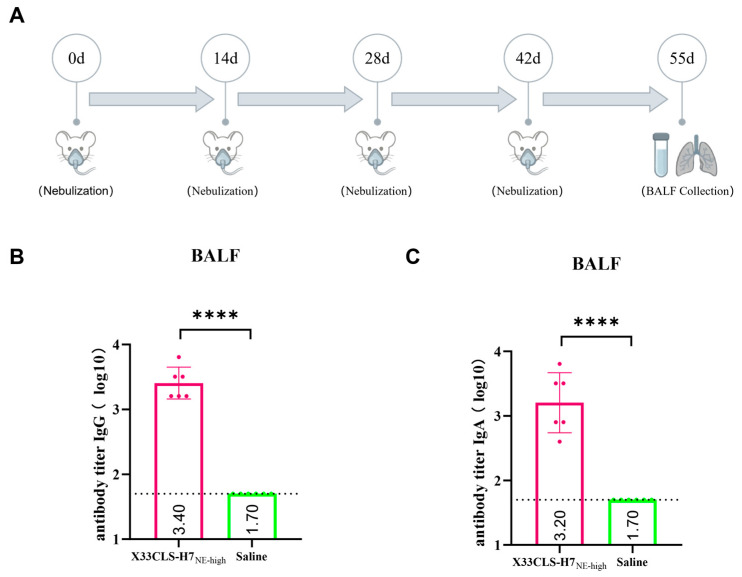
High-dose nebulized X33CLS-H7 induced lower-airway IgG and IgA responses in BALF. (**A**) Schematic diagram of the high-dose nebulized immunization schedule and BALF collection. Mice received nebulized immunization on days 0, 14, 28, and 42, and BALF was collected on day 55. (**B**) BALF HA7-specific IgG titers in mice receiving high-dose nebulized X33CLS-H7 or saline control after the final immunization. (**C**) BALF HA7-specific IgA titers in mice receiving high-dose nebulized X33CLS-H7 or saline control after the final immunization. In ELISA panels, titers are shown as log10 reciprocal endpoint titers. The dotted horizontal line indicates the lower limit of detection, with values below the starting dilution assigned 1:50, corresponding to log10 = 1.70, for plotting. Data are presented as mean ± SD. Statistical analysis was performed using unpaired *t*-tests for between-group comparisons. Statistical significance is shown in the figure (**** *p* < 0.0001).

To evaluate lower-airway antibody responses, bronchoalveolar lavage fluid (BALF) was collected from the X33CLS-H7_NE-high_ group two weeks after the final immunization, with the saline group serving as the negative control (Figure 6A). BALF analysis was restricted to the high-dose nebulized group because lower-airway humoral responses after aerosol delivery were prioritized in the present study.

In the X33CLS-H7_NE-high_ group, the BALF HA7-specific IgG titer was approximately 2.51 × 10^3^, which was significantly higher than that in the saline control group (*p* < 0.0001) (Figure 6B). Likewise, the BALF HA7-specific IgA titer was approximately 1.58 × 10^3^, and was also significantly higher than that in the saline control group (*p* < 0.0001) (Figure 6C). These results indicate that high-dose nebulized X33CLS-H7 induced detectable HA7-specific IgG and IgA responses in the bronchoalveolar compartment.

### 3.6. Safety Evaluation

**Figure 7:**
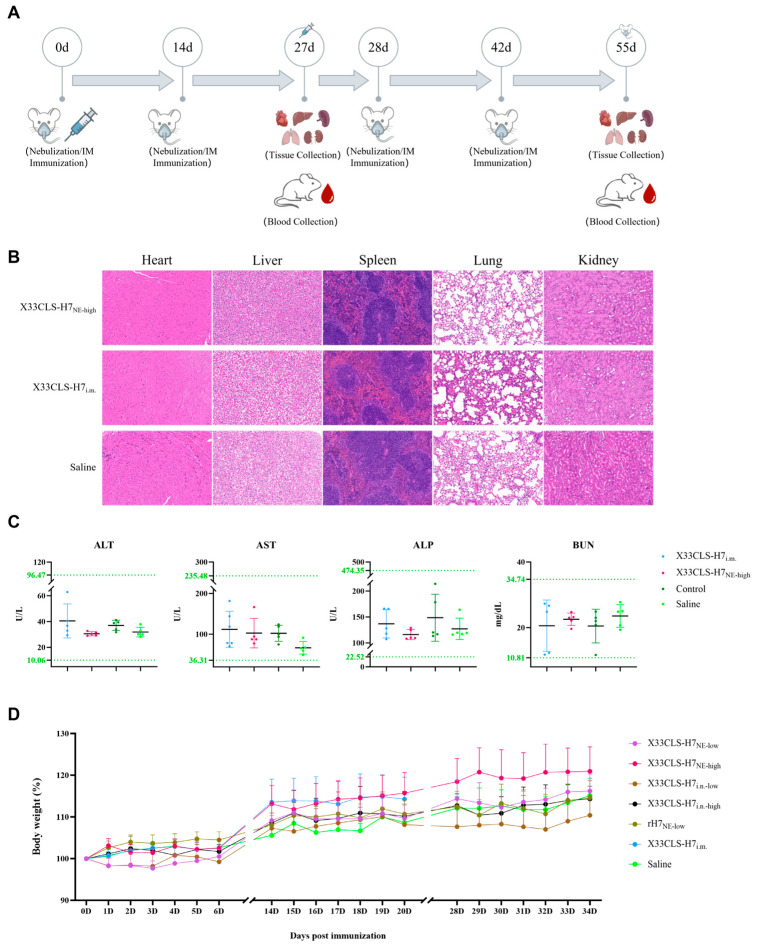
Safety evaluation of X33CLS-H7 immunization in mice. (**A**) Schematic diagram of the safety evaluation schedule. (**B**) Representative hematoxylin and eosin (H&E)-stained sections of the heart, liver, spleen, lung, and kidney. (**C**) Serum biochemistry analysis of alanine aminotransferase (ALT), aspartate aminotransferase (AST), alkaline phosphatase (ALP), and blood urea nitrogen (BUN) in mice from the indicated groups. The dotted lines indicate the reference ranges. (**D**) Longitudinal body weight changes in mice receiving different immunization regimens during the study period. Data are presented as mean ± SD.

Throughout the observation period mice remained in stable general condition, and body weight trajectories were broadly comparable to those of the saline control group, with no abnormal weight loss or growth retardation suggestive of overt toxicity (Figure 7D). Histopathological examination showed no clear vaccine-associated tissue injury or obvious inflammatory lesions in the heart, liver, spleen, lung, or kidney. Specifically, tissues from the X33CLS-H7_i.m._ group were collected on day 27, whereas tissues from the X33CLS-H7_NE-high_ and saline control groups were collected on day 55; neither group showed evident pathological abnormalities relative to the saline control group (Figure 7B). Likewise, serum biochemistry was also assessed to evaluate potential systemic toxicity or organ stress. ALT, AST, ALP, and BUN values remained within the reference ranges and were not markedly different from those of the control groups (Figure 7C). Collectively, these observations did not reveal overt local or systemic safety signals associated with X33CLS-H7 immunization under the tested intramuscular or respiratory mucosal delivery conditions.

## 4. Discussion

Establishing efficient immune protection against respiratory pathogens such as influenza A virus remains a major challenge in both veterinary and public health settings [17]. In this present study, X33CLS-H7, a clarified cell-lysate supernatant prepared from glycoengineered *P. pastoris*, retained H7-associated antigenicity and hemagglutination activity despite not being purified into a single recombinant HA protein. This was supported by Western blot detection of the target HA7 band and by measurable hemagglutination activity. Functionally, X33CLS-H7 induced antibody responses through multiple routes of administration. Two intramuscular immunizations rapidly elicited the strongest systemic responses, including high levels of serum HA7-specific IgG, robust HI activity, and detectable pseudovirus neutralizing antibodies. In contrast, respiratory mucosal delivery showed more route- and dose-dependent characteristics. The high-dose nebulized regimen induced the strongest systemic antibody profile among the respiratory delivery groups and generated detectable HA7-specific IgG and IgA responses in BALF, whereas intranasal instillation also induced serum IgG, IgA, and HI responses, although at lower overall magnitudes than high-dose nebulization. In parallel, body-weight monitoring, histopathology examination, and serum biochemistry analysis did not reveal overt local or systemic toxicity signals under the experimental conditions tested.

The H7N9 pseudovirus neutralization assay extends the chain of evidence from antibody binding and hemagglutination inhibition to a functional readout of viral entry inhibition. The intramuscular X33CLS-H7 group showed the highest log10 NT_50_ value, while the high-dose nebulized group, although lower than the intramuscular group, still exhibited a significantly higher NT_50_ value than the saline control group. These results indicate that antibodies induced by X33CLS-H7 were not limited to HA recognition or inhibition of hemagglutination, but were also capable of interfering with H7N9 pseudovirus entry into target cells. Importantly, HI activity and pseudovirus neutralization should not be interpreted as identical endpoints. HI primarily reflects antibody-mediated blockade of HA-receptor binding, whereas pseudovirus neutralization captures a broader functional outcome related to inhibition of viral entry. Previous H7N9 pseudovirus studies have shown that pseudovirus-based neutralization assays correlate with HI and live-virus microneutralization assays, supporting their use as practical functional serological endpoints for H7N9 vaccine evaluation [18]. Nevertheless, because the intramuscular and high-dose nebulized groups differed in both immunization procedure and sampling schedule, the higher NT_50_ value observed after intramuscular immunization should not be overinterpreted as definitive superiority of this route. Rather, the data suggest that both systemic injection and respiratory nebulization can induce functional antibody responses, with intramuscular administration being more efficient in generating serum neutralizing activity under the present experimental conditions.

A major finding of this study is that respiratory mucosal delivery induced not only systemic antibody responses but also detectable lower-airway IgA, particularly after high-dose nebulization. This observation is consistent with the established concept that mucosal immunization is well suited to generate local immune responses at the site of pathogen entry [8]. Such responses are especially relevant for influenza control, because immunity at the respiratory mucosa may contribute more directly to limiting infection establishment and viral shedding than serum antibody responses alone. Human influenza challenge studies have shown that pre-existing nasal IgA is associated with reduced duration of viral shedding, and that mucosal and systemic immune responses can be partially compartmentalized [19]. Similarly, studies using intramuscular inactivated influenza vaccines have indicated that strong systemic antibody responses do not necessarily translate into equally robust mucosal immunity [20]. In this context, the stronger serum neutralization induced by intramuscular X33CLS-H7 and the detectable BALF IgG and IgA induced by high-dose nebulized X33CLS-H7 should be interpreted as evidence of route-dependent immune spatial organization rather than as mutually conflicting outcomes.

From a production perspective, X33CLS-H7 also has practical advantages. Conventional inactivated avian influenza vaccines require propagation and handling of live virus under appropriate containment conditions and are relatively labor- and cost-intensive to manufacture and administer [21]. By contrast, *P. pastoris* is a well-established recombinant protein expression platform with advantages in high-density fermentation, scalability, and process adaptability [13]. Glycoengineering further improves its suitability for producing glycoprotein vaccine antigens by enabling more mammalian-like N-glycosylation patterns, which may favor structural stability and epitope presentation [22]. Together with previous evidence showing that H7 expressed in glycoengineered *P. pastoris* can preserve relevant epitopes and protect mice against H7N9 challenge [14], our results support the possibility that a simplified clarified-lysate format can retain biologically relevant HA-associated immunogenicity while reducing downstream manufacturing complexity. The comparable endpoint IgG, IgA, and HI responses induced by purified rH7 and X33CLS-H7 under matched low-dose nebulized conditions further suggest that the CLS format did not compromise antigenicity under the tested conditions. Therefore, the primary value of X33CLS-H7 may lie not necessarily in achieving the highest serum antibody magnitude, but in its manufacturing simplicity, potential cost efficiency, and compatibility with respiratory mucosal delivery strategies.

However, the clarified lysate format also introduces important interpretive limitations. X33CLS-H7 was evaluated as a clarified cell-lysate antigen preparation rather than as purified HA, and residual yeast-derived components may have contributed to immunostimulation. Although the assay design and comparator analyses support predominantly HA7-directed immunogenicity, the specific contribution of host-derived components was not fully dissected in the present study. Future studies should therefore include direct comparisons among purified rH7, X33CLS-H7, and host-component-depleted preparations, together with batch-to-batch consistency testing and detailed characterization of residual yeast-derived components. These experiments will be essential for distinguishing the contribution of the H7 antigen itself from potential innate immune stimulation associated with the CLS matrix.

Several additional limitations should be acknowledged. First, this study was conducted in mice rather than in target avian species. Although the mouse model is useful for preliminary immunogenicity and safety evaluation, chickens and ducks are more relevant hosts for assessing the translational potential of avian influenza vaccine candidates. Second, although pseudovirus neutralization was included in the present study, the current dataset does not include live-virus microneutralization, challenge protection, viral shedding, or transmission outcomes, all of which are important endpoints for functional and translational evaluation of veterinary influenza vaccines [21,23]. Third, for nebulization, the nominal antigen amount reflected the dose loaded into the nebulizer rather than the directly quantified deposited respiratory dose. Device dead volume, aerosol dispersion, chamber retention, and exhalation may all influence the actual delivered dose. Fourth, for intranasal instillation, dose escalation was achieved by increasing the administered volume from the same antigen stock, so dose and volume effects could not be fully disentangled. Fifth, BALF antibodies were assessed only in the high-dose nebulized group because nebulization was prioritized as the principal mucosal route of practical interest. Consequently, lower-airway responses could not be directly compared between nebulized and intranasal delivery in the present study. Sixth, the present study focused mainly on humoral immune readouts and did not systematically evaluate the cellular components of the immune response. Therefore, the contribution of antigen-specific T-cell responses to immunogenicity and potential protection, particularly in local respiratory compartments, remains to be defined.

Future studies should evaluate X33CLS-H7 in chickens or ducks, with particular emphasis on challenge protection, viral load, shedding duration, and contact transmission. Quantitative analysis of aerosol particle size, delivery efficiency, and deposited respiratory dose will also be necessary to optimize nebulized administration. Spray-based mucosal immunization may be more compatible with mass poultry vaccination [24], but its practical value will depend on whether group-level delivery can achieve consistent and sufficient respiratory exposure. Alternative prime-boost strategies, such as intramuscular priming followed by mucosal boosting, may further improve the balance between systemic HI/neutralizing responses and respiratory mucosal immunity [25]. Ultimately, the value of this candidate will depend on whether the immunological advantages observed in the present mouse model can be translated into reduced infection, shedding, and transmission under relevant avian host conditions [19].

## 5. Conclusions

In conclusion, the glycoengineered *P. pastoris*-derived X33CLS-H7 antigen can be produced as a simplified clarified cell-lysate supernatant while retaining relevant H7 antigenicity and hemagglutination activity. In mice, X33CLS-H7 induced robust systemic antibody responses after intramuscular immunization and measurable systemic and lower-airway antibody responses after respiratory mucosal delivery, particularly following high-dose nebulization. Pseudovirus neutralization further indicated that X33CLS-H7-induced antibodies could inhibit H7N9 pseudovirus entry. No overt local or systemic toxicity signals were observed under the tested conditions. These findings support X33CLS-H7 as a simplified and scalable H7N9 antigen candidate with multi-route delivery potential. Further evaluation in target avian species, including challenge protection, viral shedding, transmission, and aerosol deposition studies, is warranted.

## Figures and Tables

**Table 1:** Mouse immunization protocol. Doses for X33CLS-H7 are expressed as estimated HA-equivalent inputs normalized by hemagglutination activity rather than as absolute purified HA mass. For nebulized groups, the listed dose represents the amount loaded into the nebulizer system rather than a directly measured deposited respiratory dose. For intranasal groups, dose escalation was achieved by increasing the administered volume from the same antigen stock preparation. NE, nebulized inhalation; i.n., intranasal instillation; i.m., intramuscular injection.

Group	Route	Antigen	Estimated HA-Equivalent Input	Adjuvant	Volume	*n*
rH7_NE-low_	NE	rH7	36 µg	—	200 µL	6
X33CLS-H7_NE-low_	NE	X33CLS-H7	36 µg	—	200 µL	6
X33CLS-H7_NE-high_	NE	X33CLS-H7	144 µg	—	800 µL	6
X33CLS-H7_i.n.-low_	i.n.	X33CLS-H7	3.6 µg	—	20 µL	6
X33CLS-H7_i.n.-high_	i.n.	X33CLS-H7	10.8 µg	—	60 µL	6
X33CLS-H7_i.m._	i.m.	X33CLS-H7	18 µg	—	100 µL	6
X33CLS-H7_i.m._	i.m.	X33CLS-H7	9 µg	—	100 µL	6
X33CLS-H7_i.m._	i.m.	X33CLS-H7	9 µg	CFA (1:1)	100 µL	6
X33CLS-H7_i.m._	i.m.	X33CLS-H7	9 µg	IFA (1:1)	100 µL	6
X33CLS-H7_i.m._	i.m.	X33CLS-H7	9 µg	AS03 (1:1)	100 µL	6
CFA control	i.m.	—	—	CFA	100 µL	6
Control	i.m.	X33CLS	—	—	100 µL	6
Saline	NE	—	—	—	200 µL	6

**Table 2:** Summary of experimental groups, immunization regimens, and principal evaluation readouts.

Group	Adjuvant	Estimated HA-Equivalent Input	Immunization Schedule	Principal Readouts
rH7_NE-low_	—	36 µg	0d, 14d, 28d, 42d	Serum HA7-specific IgG, IgA, HI
X33CLS-H7_NE-low_	—	36 µg	0d, 14d, 28d, 42d	Serum HA7-specific IgG, IgA, HI
X33CLS-H7_NE-high_	—	144 µg	0d, 14d, 28d, 42d	Serum HA7-specific IgG, IgA, HI; pseudovirus neutralization titer (NT_50_); BALF HA7-specific IgG and IgA; histopathology; serum biochemistry
X33CLS-H7_i.n.-low_	—	3.6 µg	0d, 14d, 28d, 42d	Serum HA7-specific IgG, IgA, HI
X33CLS-H7_i.n.-high_	—	10.8 µg	0d, 14d, 28d, 42d	Serum HA7-specific IgG, IgA, HI
X33CLS-H7_i.m._	—	18 µg	0d, 14d	Serum HA7-specific IgG, HI; pseudovirus neutralization titer (NT_50_); histopathology; serum biochemistry
X33CLS-H7_i.m._	—	9 µg	Single dose	HI comparison in adjuvant study
X33CLS-H7_i.m._	CFA (1:1)	9 µg	Single dose	HI comparison in adjuvant study
X33CLS-H7_i.m._	IFA (1:1)	9 µg	Single dose	HI comparison in adjuvant study
X33CLS-H7_i.m._	AS03 (1:1)	9 µg	Single dose	HI comparison in adjuvant study
CFA control	CFA	—	Single dose	HI comparison in adjuvant study
Control	—	—	Matched to i.m. regimen	Negative control for serum antibody/HI comparisons
Saline	—	—	Matched to NE regimen	Negative control for serum antibody, BALF, histopathology, serum biochemistry, and pseudovirus neutralization titer (NT_50_)

**Table 3:** Endpoint HI geometric mean titers and corresponding 95% confidence intervals in intramuscular immunization groups. HI GMTs and 95% confidence intervals were calculated from log10-transformed individual HI endpoint titers and then back-transformed to the reciprocal titer scale. Values of 10 were used for samples at the lower limit of detection.

Group	Endpoint Day	Individual HI Titers	HI GMT	95% CI
CFA control	Day 13	10, 10, 10, 10, 10, 10	1:10	1:10–1:10
Control	Day 27	10, 10, 10, 10, 10, 10	1:10	1:10–1:10
X33CLS-H7_i.m._ + CFA, 9 µg	Day 13	160, 80, 80, 40, 80, 40	1:71	1:41–1:123
X33CLS-H7_i.m._ + IFA, 9 µg	Day 13	80, 80, 80, 80, 40, 40	1:63	1:44–1:92
X33CLS-H7_i.m._ + AS03, 9 µg	Day 13	320, 160, 320, 160, 80, 320	1:202	1:111–1:365
X33CLS-H7_i.m._ + —, 9 µg	Day 13	10, 40, 20, 20, 40, 80	1:28	1:13–1:61
X33CLS-H7_i.m._ + —, 18 µg	Day 13	80, 40, 20, 20, 40, 80	1:40	1:21–1:77
X33CLS-H7_i.m._ + —, 18 µg	Day 27	2560, 1280, 2560, 1280, 1280, 1280	1:1622	1:1108–1:2348

**Table 4:** Endpoint HI geometric mean titers and corresponding 95% confidence intervals in respiratory mucosal immunization groups at day 55. HI GMTs and 95% confidence intervals were calculated from log10-transformed individual HI endpoint titers and then back-transformed to the reciprocal titer scale. Values of 10 were used for samples at the lower limit of detection.

Group	Route	Individual HI Titers	HI GMT	95% CI
Saline	NE	10, 10, 10, 10, 10, 10	1:10	1:10–1:10
rH7_NE-low_	NE	160, 10, 20, 10, 40, 40	1:28	1:9–1:85
X33CLS-H7_NE-low_	NE	20, 10, 10, 20, 40, 80	1:22	1:10–1:53
X33CLS-H7_NE-high_	NE	640, 160, 640, 320, 320, 1280	1:457	1:211–1:971
X33CLS-H7_i.n.-low_	i.n.	40, 160, 320, 80, 20, 160	1:90	1:31–1:262
X33CLS-H7_i.n.-high_	i.n.	160, 320, 320, 640, 640, 40	1:254	1:85–1:759

## Data Availability

The raw data supporting the conclusions of this article will be made available by the authors, without undue reservation.
